# Predicting Cochlear Synaptopathy in Mice with Varying Degrees of Outer Hair Cell Dysfunction Using Auditory Evoked Potentials

**DOI:** 10.1007/s10162-025-01015-x

**Published:** 2025-12-13

**Authors:** Brad N. Buran, Seán Elkins, Wenxuan He, Sarah Verhulst, Naomi F. Bramhall

**Affiliations:** 1 Oregon Hearing Research Center, Oregon Health & Science University, Portland, OR USA; 2 Hearing Technology @ WAVES, Department of Information Technology, Ghent University, Zwijnaarde, Belgium; 3 VA RR&D National Center for Rehabilitative Auditory Research, VA Portland Health Care System, Portland, OR USA; 4 Department of Otolaryngology – Head & Neck Surgery, Oregon Health & Science University, Portland, OR USA

**Keywords:** Cochlear synaptopathy, Envelope following response (EFR), Auditory brainstem response (ABR), Age-related hearing loss, Noise-induced hearing loss, Cochlear deafferentation

## Abstract

**Purpose:**

Although human temporal bones suggest that cochlear synapse numbers decline with age and noise exposure, no validated diagnostic method exists. In animal models, cochlear synaptopathy is associated with reduced auditory brainstem response (ABR) wave 1 amplitude and envelope following response (EFR) magnitude for a sinusoidally amplitude modulated (SAM) tone. However, measuring SAM EFR at the optimal modulation frequency (1000 Hz) is difficult in humans. A rectangular amplitude modulated (RAM) tone may be more sensitive to synaptopathy, but this has not been validated in animals. In addition, because synaptopathy likely co-occurs with outer hair cell dysfunction (OHC), a diagnostic assay needs to be robust to abnormal auditory thresholds. The objective of this study was to evaluate the relative ability of ABR and EFR measures to predict synapse numbers in mice with varying degrees of synaptopathy and OHC dysfunction.

**Methods:**

Distortion product otoacoustic emissions (DPOAEs), ABR, SAM EFR, and RAM EFR were recorded from 57 mice with a range of auditory thresholds and degrees of synaptopathy. Cross-validation was used to compare the relative ability of linear regression models incorporating different measures to predict synapse numbers. Predictions were confirmed histologically.

**Results:**

RAM EFR modulated at 1000 Hz was the single best predictor of synapse numbers for broad synapse loss across frequency, while combining RAM EFR with ABR further improved predictions. In contrast, ABR best predicted focal synaptopathy. Incorporating DPOAEs improved predictions for EFR, but not ABR.

**Conclusion:**

RAM EFR, ABR, and DPOAEs should be used in the future when predicting synapse numbers.

**Supplementary Information:**

The online version contains supplementary material available at 10.1007/s10162-025-01015-x.

## Introduction

Cochlear synaptopathy, which is the loss of the synapses between the inner hair cells (IHCs) and the afferent auditory nerve fibers, results from aging and noise exposure in animal models [1, 2]. Human temporal bones suggest that synaptopathy also occurs in humans with age [3] and military/occupational noise exposure [4]. Due to the high predicted prevalence of synaptopathy, the development of a diagnostic measure is of high importance. Although synaptopathy can only be confirmed through post-mortem analysis, two auditory evoked potentials have been identified in mice as possible non-invasive indicators of synaptopathy; the auditory brainstem response (ABR) wave 1 amplitude [1, 2] and the envelope following response (EFR) [5, 6].

It has been suggested that ABR wave 1 amplitude may underestimate synaptopathy because the compound action potential (CAP), similar to the ABR wave 1 amplitude measurement but acquired with an electrode placed on the round window niche, appears to be unaffected by the loss of low spontaneous rate (SR) auditory nerve fibers [7], presumably because the CAP is an onset response and low SR fibers have poorer onset coding than medium and high SR fibers [8]. This is problematic because low SR fibers appear to be particularly vulnerable to synaptopathy [9, 10]. The EFR measures the sustained response to an amplitude modulated stimulus, rather than the onset response. Computational modeling suggests that the EFR is sensitive to the loss of low SR fibers [11], which could make it a better indicator of synaptopathy than ABR wave 1 amplitude. However, translating the EFR from mouse to humans presents challenges. Higher modulation frequencies (*f*_*m*_) are thought to reflect auditory nerve function [5, 6] while lower modulation frequencies appear to be generated primarily by the brainstem [12]. In mice, the EFR is most sensitive to synaptopathy when *f*_*m*_ = ~ 1000 Hz [5]. While most studies of human synaptopathy use low modulation frequencies (*f*_*m*_ = 80–120 Hz) because high frequencies result in very small EFR magnitudes [13], McHaney et al. [14] showed that it is possible to record EFRs in young adults when *f*_*m*_ = 1024 Hz. Although the EFR has only been measured in animal models of noise- or age-related synaptopathy using a sinusoidally amplitude-modulated (SAM) stimulus, computational modeling suggests that a rectangular amplitude-modulated (RAM) stimulus may be less sensitive to OHC dysfunction due to its sharply rising envelope [15], thereby making changes in EFR magnitude more sensitive to synaptopathy. However, the sharp onset of the stimulus may make the RAM EFR more similar to the ABR, potentially resulting in decreased sensitivity to low SR fiber loss. Despite this caveat, Garrett et al. [16] showed a greater reduction in EFR magnitude compared to controls using a RAM versus a SAM stimulus (*f*_*m*_ = 100 Hz) in budgerigars with kainic acid-induced synaptopathy. Adding further to uncertainty about how to use the EFR to detect synaptopathy, the methods for calculating EFR strength have varied across human studies [e.g., 17, 18].

Two studies obtained ABR/EFR measures and synapse numbers in the same animals. Shaheen et al. [5] compared ABR and SAM EFR (*f*_*m*_ = 1000 Hz) in mice with noise-induced synaptopathy and reported larger effect sizes for SAM EFR. Parthasarathy and Kujawa [6] evaluated ABR and SAM EFR (*f*_*m*_ = 1028 Hz) in mice with age-related synaptopathy and observed larger effect sizes for the EFR at 30 kHz, but the opposite at 12 kHz. This provides modest support for the SAM EFR (*f*_*m*_ = ~ 1000 Hz). However, it is unclear how the RAM EFR compares to the SAM EFR and ABR. The best measure may depend on the synaptopathy configuration. In mice, noise-induced synaptopathy initially results in a focal loss of synapses in the 22.6–64 kHz cochlear region [1] which broadens over time to include lower frequencies [19]. Broad synapse loss also occurs in mice with age-related synaptopathy [2] and in older humans [3]. Another important consideration is how each measure is impacted by OHC dysfunction, which likely co-exists with synaptopathy in many cases.

The objective of this study was to evaluate the relative ability of evoked potential measures to predict synapse numbers in mice with varying degrees of synaptopathy and OHC dysfunction. The measures included ABR wave 1 amplitude, SAM EFR (*f*_*m*_ = 110 Hz and 1000 Hz), and RAM EFR (*f*_*m*_ = 110 Hz and 1000 Hz). Different methods of ABR and EFR processing were also investigated.

## Materials and Methods

### Experimental Design

Four groups of CBA/CaJ mice (Jackson Laboratories #000654, https://www.jax.org/strain/000654) of either sex (25 male, 32 female) were included in this study: young (*n* = 17), acute noise exposed (*n* = 13), aged (*n* = 14), and aged after noise exposure (*n* = 13). The goal was to generate a sample of mice that encompassed a wide variety of degrees of cochlear synapse loss with varying degrees of OHC damage. Mice were broadly grouped based on the pattern of synapse loss across the cochlea. Not all mice in a particular group were the same age or experienced the same noise exposure. The young mice were tested between 10 and 42 weeks of age. The acute noise exposed mice were exposed to 94 or 98 dB SPL at 8 weeks of age and were tested between 10 and 13 weeks of age. The aged mice were tested between 82 and 103 weeks of age. The aged after noise exposure mice were noise exposed to 101 dB SPL at 16 weeks of age and were tested between 80 and 104 weeks of age. Both the acute noise exposed and aged after noise exposure groups were exposed to octave-band (8 to 16 kHz) noise for 2 h. All physiological and histological measures were performed in the left ear for each mouse. Mouse data were included in the dataset even if a full set of physiological and histological measures were not available (e.g., 35% of experiments did not measure the SAM and RAM EFR modulated at 1 kHz for a carrier frequency of 32 kHz). All mice previously received a headplate surgery for awake assessment of auditory function for a separate study. Mice were excluded from the study if their DPOAE thresholds at 16 kHz were 10 dB greater than prior to the headplate surgery. Since the goal was to collect data from a sample of mice heterogenous with respect to auditory function, there were no other criteria for exclusion of mice from this study other than health reasons as recommended by a veterinarian.

### Noise Exposure

Noise exposures were performed following Kujawa and Liberman [1]. Awake mice were placed in a custom-built wire cage inside a custom-built noise exposure chamber. The cage was subdivided into six compartments with one mouse per compartment. The cage was positioned on a turntable, ensuring that all mice received a uniform noise exposure that was not affected by the development of standing waves in the chamber. The noise exposure chamber was designed as a trapezoidal prism with only the top and bottom surfaces parallel to each other. Noise exposure was controlled using a custom-written program [20]. Noise stimuli were generated digitally by drawing random samples from a uniform distribution and then bandpass filtered using a 1001 tap finite impulse response filter with a passband of 8 to 16 kHz (> 60 dB/octave slope). To ensure uniform spectral distribution of the noise, the gain of the passband was adjusted to equalize the noise. Stimuli were converted to analog (PCI-6251, National Instruments), amplified (D75-A, Crown Audio), and delivered via a 1-inch compression driver (D220 Ti, JBL Professional Loudspeakers) coupled to a horn (HM25-25, JBL Professional Loudspeakers). Prior to each noise exposure, the intensity level of the noise was calibrated to the target level using a 1/4-inch microphone (377C01 coupled to a 426B03 preamplifier and powered by a 480C02 signal conditioner, PCB Piezotronics).

### Physiological Measures

Animals were anesthetized with a ketamine (100 mg/kg) and xylazine (10 mg/kg) cocktail. Body temperature was monitored rectally and maintained using a heating pad regulated by a homeothermic temperature controller (50-7503F, Harvard Apparatus). A custom-built acoustic system consisting of two speakers and an embedded microphone [21] was positioned inside the intratragal notch just above the external acoustic pore (i.e., the opening of the ear canal). Stimuli were generated digitally using a custom data acquisition program [22], converted to analog (PXI-4461, National Instruments), amplified (SA-1, Tucker-Davis Technologies), high-pass filtered at 500 Hz using a custom-built RC circuit, and delivered to the ear via one of the speakers (CDMG15008-03A, Same Sky [formerly CUI Devices]) in the acoustic system. The embedded microphone (FG-23329-P07, Knowles Electret) was calibrated using a 1/8-inch microphone (46-DP1 powered by a 12AK, GRAS Acoustics). In-ear calibration of the speakers was performed immediately prior to each experiment using the embedded microphone. Responses to ABR and EFR stimuli were collected using needle electrodes (F-E2-12, Natus Medical) positioned at the vertex and intratragal notch with a ground near the tail. Responses were amplified (50,000 ×), band-pass filtered from 10 to 10,000 Hz (P511, Grass Instruments), and digitized (PXI-4461, National Instruments) for further analysis. The full set of physiological measurements were collected in a single session.

ABR stimuli consisted of 5 ms tone pips (0.5 ms cosine-squared rise-fall ramp with 4 ms steady-state). A full stimulus train consisting of a single presentation of each of seven frequencies (5.6 to 45.2 kHz in half-octave steps) and 15 levels (10 to 80 dB SPL in 5 dB steps) was constructed with individual stimuli presented at a rate of 81 per second. Since the ordering of frequencies and levels was arranged in the interleaved ramp design described in Buran et al. [21], which leverages auditory nerve fiber tuning to minimize adaptation, the effective stimulus rate was 11.6 per second for each frequency. ABRs were presented in alternating polarity and a total of 512 artifact-free trials were collected (256 for each polarity). Ensemble averages of ABR waveforms were bandpass filtered at 300 to 3000 Hz and the peak for wave 1 was initially assigned using semi-automated peak-picking software [23] and then reviewed visually by an experienced rater. Raters were blinded to the experimental group. ABR thresholds were identified using an automated algorithm [24].

**Fig. 1:**
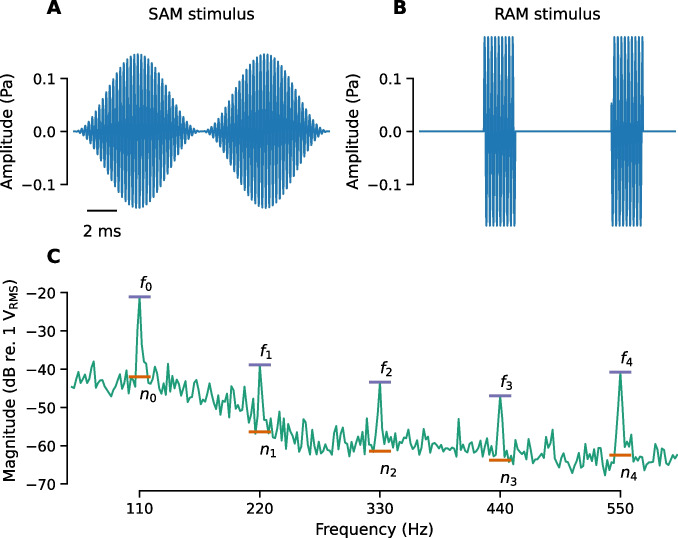
Schematic showing EFR stimuli and methods for analyzing the EFR response. Waveforms for SAM (**A**) and RAM (**B**) stimuli modulated at 110 Hz. Stimuli are scaled so that the overall RMS for both stimuli is 70 dB SPL. **C** Magnitude spectrum of the EFR response to a RAM stimulus modulated at 110 Hz illustrating the features that are used in different methods of calculating EFR magnitude, including the noise floor (*n*_0_–*n*_4_), the modulation frequency (*f*_0_) and the first five multiples of the modulation frequency (*f*_0_–*f*_4_). EFR magnitude can either be extracted at *f*_0_ only or by summing *f*_0–4_ and can be expressed either as an absolute value (*f*_0_, *f*_0–4_) or relative to the noise floor (*f*_0_ dB SNR, *f*_0–4_ dB SNR)

EFR stimuli consisted of 500 ms amplitude-modulated carrier tones with an inter-stimulus interval jittered uniformly between 100 and 120 ms for an average stimulus rate of 1.64/s. Carrier frequencies were 16 or 32 kHz with an overall stimulus level of 70 dB SPL. Stimuli were either sinusoidally (SAM) or rectangular amplitude modulated (RAM) at 110 or 1000 Hz. For SAM tones, the modulation depth was 100%. For RAM tones, stimuli were designed as described in Vasilkov et al. [15] with a modulation depth of 100%, duty cycle of 25%, and a 2.5% Tukey window applied to the onset and offset of each individual cycle of the RAM tone to provide gradual transitions. Stimuli were presented in alternating polarity and a total of 128 trials were collected (64 for each polarity). As illustrated in Fig. 1, different methods of analyzing the EFR data were evaluated due to differences in EFR analysis method across previous studies: (1) compute the magnitude at the modulation frequency (*f*_0_); (2) sum the magnitude of the first five multiples of the modulation frequency (*f*_0–4_); (3) compute the signal-to-noise ratio (SNR) of the at the modulation frequency relative to the noise floor (f_0_ dB SNR); (4) sum the magnitude of the first five multiples of the modulation frequency relative to the noise floor (f_0–4_ dB SNR); and (5) compute the phase locking value (PLV). For *f*_0–4_, the values were computed as $20\cdot\log_{10}\sum\nolimits_{i=0}^4f_i$ and for *f*_0–4_ dB SNR, the values were computed $20\cdot {\mathrm{log}}_{10}{\Sigma }_{i=0}^{4}\frac{{f}_{i}}{{n}_{i}}$ where ${f}_{i}$ is the amplitude of the $i$ th harmonic and ${n}_{i}$ is the amplitude of the noise floor surrounding the $i$ th harmonic. Both ${f}_{i}$ and ${n}_{i}$ are in units of *V*_rms_. Although it may be considered more correct to sum the powers of the harmonics using the formula $10\cdot\log_{10}\sum\nolimits_{i=0}^4f_i^2$, this approach results in a value that is strongly biased towards the frequency components with the largest magnitudes and lowest noise floors, and we found the exact method did not alter our results (Supplemental Data Fig. 1).

For all methods, EFR magnitude and phase was calculated similarly to the bootstrapping approach described in Zhu et al. [17]. In this approach, 128 trials were drawn with replacement, averaged, and the magnitude spectrum was computed. Random draws were balanced across positive and inverted polarities (i.e., 64 trials from each polarity). This process was repeated 100 times to generate a distribution of the magnitude for each frequency bin. The average value of the distribution at the modulation frequency and the first four harmonics was used to estimate the raw EFR response. To estimate the noise floor, the magnitude in the fourth to seventh discrete Fourier transform (DFT) bin on either side of the frequency of interest was averaged for a total of eight bins.

As an indicator of OHC function, distortion product otoacoustic emissions (DPOAEs) were recorded using the embedded microphone in the acoustic system. For all DPOAE measurements, the *f*_*1*_ level (*L*_*1*_) was fixed at 10 dB higher than the *f*_*2*_ level (*L*_*2*_) and the *f*_*2*_/*f*_*1*_ ratio was fixed at 1.2. DPOAE thresholds were assessed using input–output functions at seven frequencies (5.6 to 45.2 kHz in half-octave steps). For each frequency, the *f*_*2*_ level was swept from 10 to 80 dB SPL in 5 dB steps. Threshold was defined as the *f*_*2*_ level at which the DPOAE level was 0 dB SPL. To parallel the DPOAE measurements often used in human studies of synaptopathy, the DPOAE levels corresponding to *L*_*2*_ values of 40 and 55 dB SPL for *f*_*2*_ = 16 and 32 kHz were also used in the analyses.

### Histology

Immediately following the physiological measurements, mice were deeply anesthetized, decapitated, and the cochleae extracted for histology. A small hole was made in the apex of the cochleae and 4% paraformaldehyde in phosphate-buffered saline (PBS) at pH 7.3 was perfused through the round window. Cochleae were post-fixed for 2 h at room temperature and then decalcified in 10% ethylenediaminetetraacetic acid (EDTA) for 3–4 days. Once sufficiently decalcified, the cochleae were dissected into five pieces for whole mount immunostaining. Prior to immunostaining, the dissected pieces were cryoprotected in 30% sucrose and then permeabilized using a freeze–thaw step. If immunolabeling could not be initiated immediately, the cochlear pieces were stored at − 80 °C after the initial freezing step. Once thawed, cochlear pieces were rinsed in PBS, blocked with 5% normal horse serum (NHS) in PBS for one hour with 0.3% Triton-X added to further permeabilize the tissue. Primary antibodies were diluted in a solution of 1% NHS in PBS with 0.3% Triton-X. Cochlear pieces were incubated overnight at 37 °C with rabbit anti-MyosinVIIa (dilution of 1:1000, Proteus Biosciences Cat# 25–6790, RRID:AB_10015251), mouse IgG1 anti-CtBP2 (dilution of 1:200, BD Biosciences Cat# 612,044, RRID:AB_399431), and mouse IgG2a Rabbit anti-GluR2 (dilution of 1:2000, Millipore Cat# MAB397, RRID:AB_2113875). Following a wash in PBS, cochleae were incubated for one hour at 37 °C with goat anti-mouse IgG1 AF568 (Thermo Fisher Scientific Cat# A-21124, RRID:AB_2535766), goat anti-mouse Ig2a AF488 (Thermo Fisher Scientific Cat# A-21131, RRID:AB_2535771), and goat anti-rabbit AF647 (Thermo Fisher Scientific Cat# A-21245, RRID:AB_2535813). All secondary antibodies were used at a dilution of 1:1000 in 1% NHS plus 0.3% Triton-X. Following a wash in PBS, the signal was amplified by a second incubation in a freshly-made batch of the same set of secondary antibodies for 1 h at 37 °C. Pieces were washed in PBS and then labeled with 4’,6-diamidino-2-phenylindole (DAPI; Invitrogen D1306) at a dilution of 1:5000. A final wash in PBS was applied prior to mounting and coverslipping the pieces using ProLong Diamond Antifade Mountant (Thermo Fisher P36965).

A cochlear frequency map was computed using a custom program [25] that translates distance along the cochlear partition into frequency using the Greenwood function for mouse [26]. Confocal *z*-stacks were acquired for each ear at seven frequencies (5.6 to 45.2 kHz in half-octave steps) using a 1.4 NA 63 × oil-immersion objective on either a Zeiss LSM 980 or a Leica TCS SP5 confocal. CtBP2 puncta were identified using Imaris (Oxford Instruments). Assisted by custom software, identified CtBP2 puncta were inspected to identify all CtBP2 puncta paired to a closely apposed glutamate receptor patch (GluR2). Raters were blinded to the experimental group. The percent of CtBP2 puncta identified as orphans (i.e., not paired to a closely apposed GluR2 patch) are shown in Supplemental Data Fig. 2.

All procedures were approved by OHSU’s Institutional Animal Care and Use Committee and conducted in accordance with guidelines from the Office of Laboratory Animal Welfare at the National Institutes of Health. ARRIVE (Animal Research: Reporting of In Vivo Experiments) guidelines were followed in the preparation of this report.

### Statistical Analysis

Due to time constraints imposed by the anesthesia, EFR data was only collected for 16 and 32 kHz carriers. Thus, the analysis of the physiological and histological data only include data corresponding to 16 and 32 kHz stimuli or cochlear regions. Data was collected only from the left ear of each mouse to minimize test session duration. Correlations between each of the measures were assessed using the Pearson correlation coefficient from the Python scipy library [27] and confidence intervals computed using the Fisher transformation. The Bonferroni corrected confidence interval was computed as $\left(1-\frac{\left(1-\frac{c}{100}\right)}{n}\right)\cdot 100$ where $c$ is the desired confidence interval (95%) and $n$ is the number of comparisons made [28].

To test the relative ability of each evoked potential measure to predict synapse numbers, we constructed linear regression models based on various combinations of the evoked potential and DPOAE measures, ${y}_{f,i}={\beta }_{0}+{\beta }_{1}\cdot {\mathrm{M}}_{f,i}+{\beta }_{2}\cdot {\xi }_{f,i}+{\epsilon }_{f,i}$.

The number of synapses at the cochlear region corresponding to frequency $f$ in ear $i$ is represented by ${y}_{f,i}$. The frequency and ear-specific evoked potential measure is represented by ${\mathrm{M}}_{f.i}$, the frequency and ear-specific DPOAE measure is represented by ${\xi }_{f,i}$, and the residual error term is represented by ${\epsilon }_{f,i}$. When testing $p$ combinations of evoked potential measures (e.g., ABR and EFR), the model was expanded to ${y}_{f,i}={\beta }_{0}+\sum_{j=1}^{p}{\beta }_{1,j}\cdot {\overline{\mathrm{M}} }_{j,f,i}+{\beta }_{2}\cdot {\xi }_{f,i}+{\epsilon }_{f,i}$. In this equation, evoked potential measure $j$ of frequency $f$ in ear $i$ is represented by ${\overline{\mathrm{M}} }_{j,f,i}$. Models were fit using the Python statsmodels library [29].

Model performance was assessed using ten repeats of ten-fold cross-validation [30]. Cross-validation is a technique used to estimate how well a model will perform on new, unseen data. First, data from the acute noise exposed group of mice were set aside as an independent test set. This was done because these mice have distinct focal synaptopathy and we wanted to specifically evaluate the model’s performance on this condition after training. The remaining data was used for the cross-validation procedure. The data was partitioned into ten folds (i.e., groups). To ensure statistical independence between the training and validation data, all observations from a single ear were assigned to the same fold. For each of the ten iterations, one fold was held out for validation while the model was trained on the pooled data from the other nine folds. The resulting model was then used to predict synapse counts for the held-out validation data. To ensure a more stable and robust estimate of synapse prediction performance, this entire ten-fold process was repeated ten times, with the ears randomly shuffled before partitioning into folds each time. Prediction error was quantified as the root-mean-squared error (RMSE), $\sqrt{\frac{{\sum }_{k=1}^{N}{\left({y}_{k}-\widehat{{y}_{k}}\right)}^{2}}{N}}$, where ${y}_{k}$ is the $k$ th synapse count (regardless of frequency), and $\widehat{{y}_{k}}$ is the corresponding prediction. The model was evaluated on both the held-out validation sets and on the separate test set of acute noise exposed mice. The mean and standard error of the RMSE was calculated across all repeats and folds. When presenting results only from the mice with no synaptopathy or broad synaptopathy, RMSE was calculated only for the held-out validation sets. When presenting results only from the mice with focal synaptopathy (i.e., acute noise exposed), RMSE was calculated only for the separate test set of acute noise exposed mice.

The initial models evaluated a total of six different evoked potential options (no evoked potentials, ABR wave 1 amplitude at 80 dB SPL [ABR_80_], SAM EFR modulated at 110 Hz [SAM_110_], SAM EFR modulated at 1000 Hz [SAM_1000_], RAM EFR modulated at 110 Hz [RAM_110_], and RAM EFR modulated at 1000 Hz [RAM_1000_]) and four approaches for adjusting for OHC function (no adjustment, DPOAE threshold, DPOAE level at an *L*_*2*_ of 40 dB SPL [DPOAE_40_], and DPOAE level at an *L*_*2*_ of 55 dB SPL [DPOAE_55_]).

To establish a baseline for interpreting the prediction error, an intercept-only model (i.e., ${y}_{f,i}={\beta }_{0}+{\epsilon }_{f,i}$) was tested in which the predicted synapse number is the average of the observed synapse numbers in the dataset. In addition, because OHC dysfunction is highly correlated with manipulations that reduce synapse numbers (i.e., aging and noise exposure), the ability of DPOAE measures to predict synapse numbers was also evaluated (i.e., ${y}_{f,i}={\beta }_{0}+{\beta }_{2}\cdot {\xi }_{f,i}+{\epsilon }_{f,i}$). An ideal metric of synaptopathy would perform better at predicting synapse numbers than the intercept-only and the DPOAE-only models.

Due to known sex differences in ABR wave I amplitude and EFR magnitude in humans [31, 32], we evaluated whether including sex in the models, ${y}_{f,i}={\beta }_{0}+{\beta }_{1}\cdot {\mathrm{M}}_{f,i}+{\beta }_{2}\cdot {\xi }_{f,i} {+ \beta }_{3}\cdot {S}_{i}+{\epsilon }_{f,i}$ where ${\mathrm{S}}_{i}$ is 1 if the ear is from a female mouse and 0 from a male mouse, would improve synapse predictions. This analysis indicated no improvement in prediction error over models without the sex adjustment (Supplemental Data Fig. 3). For this reason, sex was not included in any further analyses.

Akaike information criterion (AIC) offers complementary information to cross-validation. In contrast to cross-validation, which estimates performance of the model on unseen data, AIC is a model selection tool that offers a relative ranking of models after penalizing for the number of parameters. AIC was calculated on each of these models after fitting to the full dataset. For this approach, the number of observations should be held constant across all models, so all combinations of ear and frequency for which we did not have a full set of observations were dropped, leaving a total of 52 ears (39 when excluding the acute noise exposed group). Since the sample size can be considered small relative to the model degree of freedom, we used a modification of the AIC that corrects for the increased risk of overfitting with small samples (AICc) [33]. For model comparison, the difference in AICc between each model and the best-performing model was calculated (ΔAICc). To interpret the relative strength of evidence for each model, we followed guidelines proposed by Burnham and Anderson [34]. Models with ΔAICc values ≤ 2 are generally considered comparable to the best performing model whereas models with ΔAICc values > 10 indicate a clear preference for the model with a lower AICc. Models with ΔAICc between 2 and 10 should not be rejected outright even though the evidence suggests that the model with a lower AICc is better.

## Results

### Wide Ranges of OHC Function and Synapse Numbers Were Represented by the Sample

**Fig. 2:**
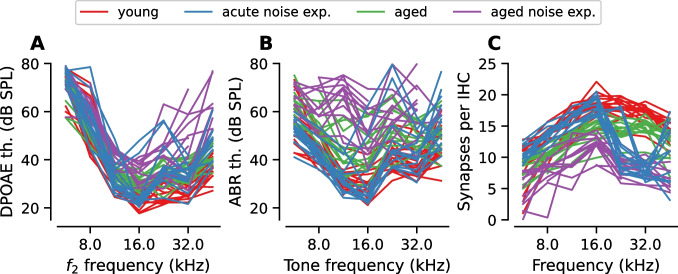
The sample included a large range of OHC dysfunction and synaptic loss. Plots show variation across individual mice from each experimental group for DPOAE thresholds (**A**), ABR thresholds (**B**), or the number of synapses per IHC across cochlear frequency (i.e., synaptogram, **C**)

Consistent with the goal of sampling mice with a heterogenous range of OHC function, DPOAE, and ABR thresholds spanned a large range of levels (Fig. 2A–B). Likewise, there was a broad range of synapse numbers across cochlear frequency in the sample (Fig. 2C). Compared to the young mice, the aged mice and the mice who were aged after noise exposure had broad synaptopathy across the entire cochlea. The degree of synaptopathy was greater for the aged mice than for the young mice and even greater for the mice who were noise exposed and then aged. In contrast, the acute noise exposed mice had focal synaptopathy where synapse numbers at cochlear regions corresponding to 16 kHz and below were comparable to synapse numbers in the young mice, while synapse numbers at the 32 kHz cochlear region were reduced ~ 50% relative to synapse numbers in the young mice.

### Relationship Between OHC Dysfunction and ABR/EFR Measurements

**Fig. 3:**
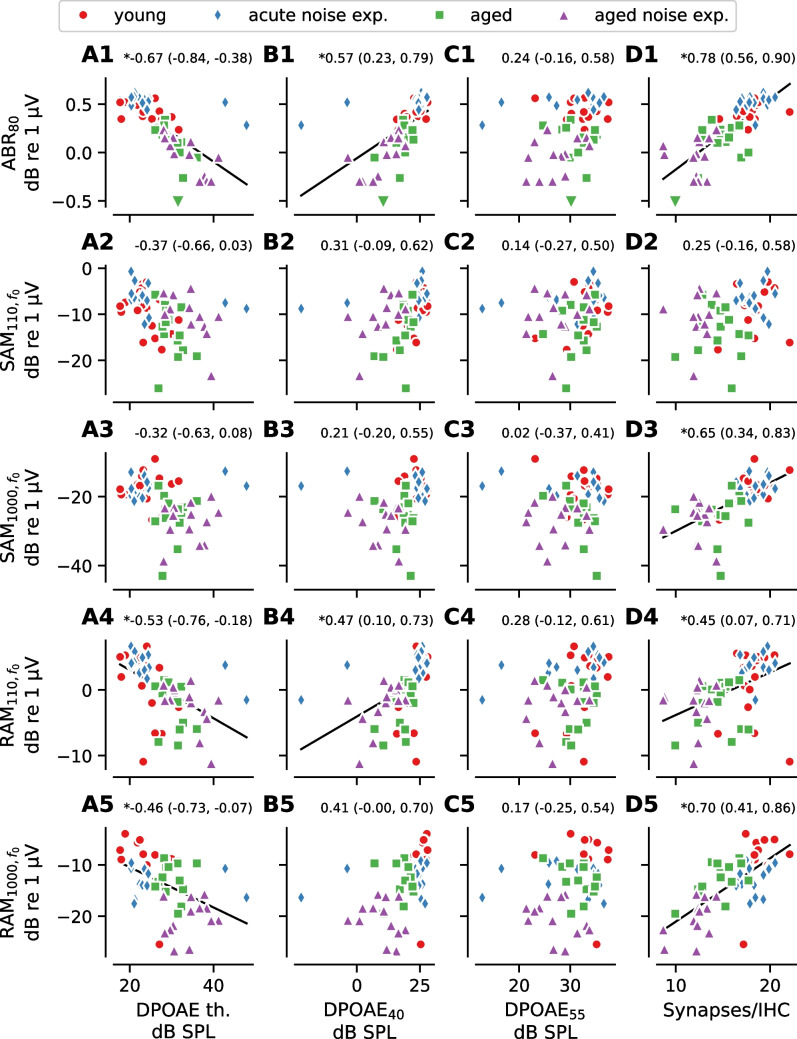
Correlations between evoked potentials and DPOAE measures or synapse counts at 16 kHz. The correlation between each of the evoked potential measures and three DPOAE measures (DPOAE threshold (**A**), DPOAE level at *L*_*2*_ = 40 dB SPL [DPOAE_40_] (**B**), DPOAE level at *L*_*2*_ = 55 dB SPL [DPOAE_55_], (**C**)) measured for a tone with *f*_*2*_ = 16 kHz or number of synapses per IHC (**D**) at the 16 kHz tonotopic region. SAM and RAM EFR magnitude were calculated as the EFR magnitude at the modulation frequency without correction for the noise floor (i.e., *f*_*0*_, see Fig. 1). For all plots, symbols indicate data from individual animals and the color/symbol type indicates the experimental group. Data limits are truncated for clarity. Downward-pointing triangles indicate data from the aged group that fall outside the view limits. Pearson’s correlation coefficients and the 95% Bonferroni-corrected (20 comparisons) confidence interval are shown above each plot. If the 95% confidence interval does not overlap with 0 (asterisk next to correlation coefficient), a regression line is shown as a visual aid. ABR_80_ = ABR wave 1 amplitude at 80 dB SPL, SAM_110_ = SAM EFR modulated at 110 Hz, SAM_1000_ = SAM EFR modulated at 1000 Hz, RAM_110_ = RAM EFR modulated at 110 Hz, RAM_1000_ = RAM EFR modulated at 1000 Hz

**Fig. 4:**
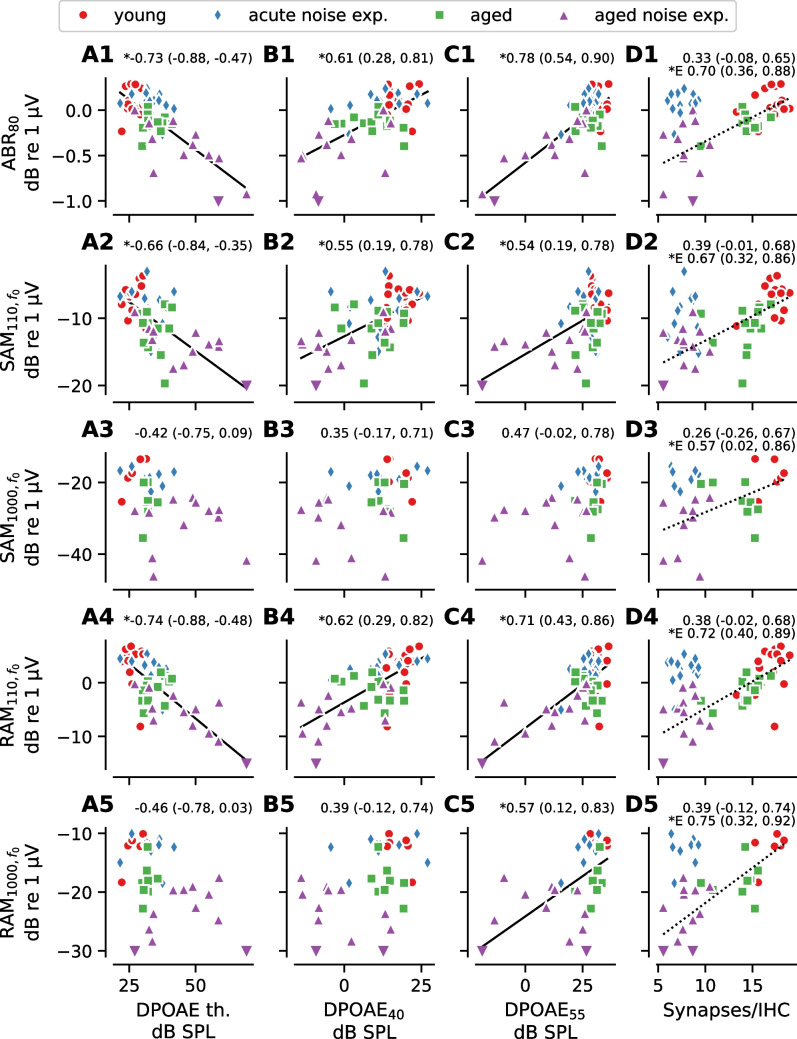
Correlations between evoked potentials and DPOAE measures or synapse counts at 32 kHz. The correlation between each of the evoked potential measures and three DPOAE measures (DPOAE threshold (**A**), DPOAE_40_ (**B**), DPOAE_55_ (**C**)) measured for a tone with *f*_*2*_ = 32 kHz or number of synapses per IHC (**D**) at the 32 kHz tonotopic region. SAM and RAM EFR magnitude were calculated as the EFR magnitude at the modulation frequency without correction for the noise floor (i.e., *f*_0_, see Fig. 1). For all plots, symbols indicate data from individual animals and the color/symbol type indicates the experimental group. Data limits are truncated for clarity. Downward-pointing triangles indicate data from the aged noise exposure group that fall outside the view limits. Pearson’s correlation coefficients and the 95% Bonferroni-corrected (20 comparisons) confidence interval are shown above each plot. If the 95% confidence interval does not overlap with 0 (asterisk next to correlation coefficient), a solid regression line is shown as a visual aid. For all panels, the correlation coefficient is computed using all ears. For synapse counts (**D**), the correlation coefficient is also computed excluding the ears that received acute noise exposure (correlation coefficient prefixed by E). If the recomputed 95% confidence interval without the acute noise exposed ears does not overlap with 0 (asterisk next to correlation coefficient), a dashed regression line is shown as a visual aid

Given that wave 1 of the ABR represents auditory nerve activity and the EFR represents the summed activity of multiple auditory nuclei, a reduction in cochlear gain due to OHC dysfunction could alter the magnitude of these evoked potential measures. To evaluate the impacts of OHC dysfunction on the ABR and EFR, correlations between each DPOAE measure and either ABR wave 1 amplitude or EFR magnitude were examined at matching frequencies (16 kHz or 32 kHz; Figs. 3 and 4). Notably, almost all evoked measures were significantly correlated with DPOAE_55_ at 32 kHz (Fig. 4C), but none were significantly correlated at 16 kHz (Fig. 3C). This may be because high level DP-grams are dominated by passive amplification (i.e., gain due to the mechanical properties of the cochlea) at 16 kHz since the active OHC amplifier is saturated at high stimulus levels relative to auditory threshold, whereas some active amplification from OHCs remains present at 32 kHz [35, 36].

### Relationship Between Synapse Numbers and ABR/EFR Measurements

With the exception of SAM_110_, all evoked potential measures were significantly correlated with synapse numbers at 16 kHz (Fig. 3D). In contrast, none of the evoked potential measures were significantly correlated with synapse numbers at 32 kHz (Fig. 4D). To assess how inclusion of the acute noise exposed mice, who had a large frequency-specific reduction in synapse numbers at 32 kHz, but near-normal evoked potential magnitudes, affected the results, we recomputed the correlations without the acute noise exposed group (dashed line in Fig. 4D). Recomputing the correlations without the acute noise exposed group resulted in stronger correlations between synapse numbers and all five evoked potential measures at 32 kHz. When considering the data at 16 kHz as well as the subset of data representing animals without acute noise exposure at 32 kHz, ABR_80_, and RAM_1000_ had the strongest correlations with synapse numbers, suggesting that ABR_80_ and RAM_1000_ may be more robust predictors of synapse numbers than the other evoked measures.

### Analysis of the Ability of Each Evoked Potential Measure to Predict Synapse Numbers

To assess which evoked potential measures have the best ability to predict cochlear synapse numbers, we used linear regression models to predict synapse numbers at cochlear frequencies of 16 and 32 kHz. Since evoked potentials can be affected by OHC dysfunction, the linear regression models optionally included an adjustment for one of the DPOAE measures. Due to the collinearity between evoked potentials and DPOAEs, the actual values of the regression coefficients (i.e., $\beta$ in the equations) cannot be interpreted when more than one predictor is included in the model. However, the predictive power of these models is not affected by multicollinearity [37]. Repeated *k*-fold cross validation was used to estimate the prediction error of each model, allowing for direct comparison of the performance of different combinations of predictors. For all predictions, only DPOAE and evoked potential data from 16 and 32 kHz was used and data from both frequencies were pooled for the model predictions (i.e., frequency-specific effects were not included).

**Fig. 5:**
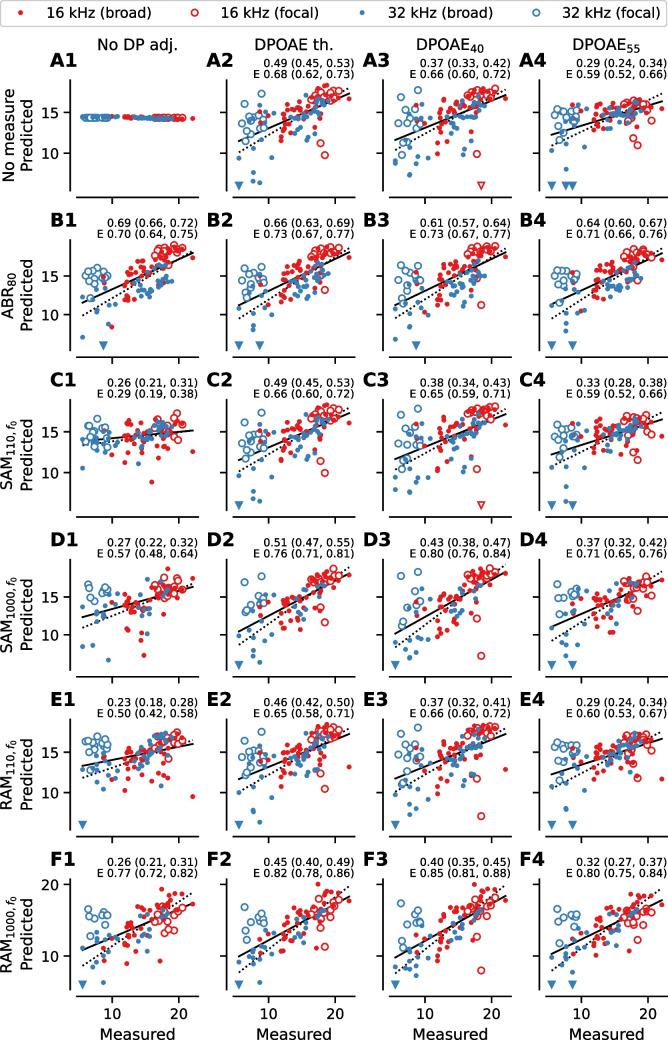
Synapse prediction model accuracy. For each linear regression model, the predicted vs. measured number of synapses per IHC is shown. Color indicates the frequency (red for 16 kHz, blue for 32 kHz), open circles indicate data from ears with acute noise exposure (focal synaptopathy), and filled circles indicate data from all other ears (no synaptopathy or broad synaptopathy). Each row shows the results of the indicated predictor without DPOAE adjustment or adjusted using one of three DPOAE measures. For reference, an intercept-only model was used to establish baseline performance (**A1**). In this model, the predicted synapse count is the average synapse count for the whole sample. The remaining subplots in the first row show the results of predicting synapses using only DPOAE metrics (**A2**–**A4**). These models establish a reference against which the ABR and EFR prediction models can be evaluated. SAM and RAM EFR magnitude were calculated as the EFR magnitude at the modulation frequency without correction for the noise floor (i.e., *f*_*0*_, see Fig. 1). Pearson’s correlation coefficients and the 95% Bonferroni-corrected (26 comparisons) confidence interval are shown above each plot. The correlation coefficient was also computed excluding ears that received acute noise exposure (correlation coefficient prefixed by E). Regression lines showing the best fit to all data (solid) or data excluding the acute noise exposure ears (dashed) are shown. For clarity, data limits were truncated and data points that are outside the visible limits are indicated by a triangle matching the color and style (open or closed) of the original symbol

All models overestimated the number of synapses at 32 kHz for the mice with focal synaptopathy due to acute noise exposure (open blue circles in Fig. 5). However, the models were generally successful at predicting synapse numbers for mice with no synaptopathy or broad synaptopathy regardless of frequency (dots in Fig. 5). When excluding the acute noise exposed group, RAM_1000_ showed the strongest correlation between predicted and actual synapse counts as compared to the other measures regardless of statistical adjustment for DPOAEs (dotted lines in Fig. 5F). When considering the full dataset (i.e., including the acute noise exposed group), the ABR_80_ showed the strongest correlation between predicted and actual synapse count regardless of statistical adjustment for DPOAEs (solid lines in Fig. 5B). This suggests that, while RAM_1000_ is the strongest predictor of broad synaptopathy, ABR_80_ is the strongest predictor of synapse number when there are cases of focal synaptopathy.

### Evaluation of Prediction Accuracy

**Fig. 6:**
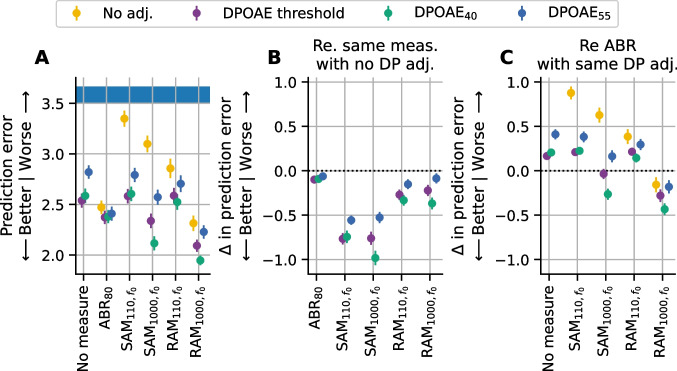
Relative ability of evoked potential models to predict synapse numbers in mice with no synaptopathy orbroad synaptopathy.** A** Root mean square error (RMSE) of each evoked potential model with and without DPOAE adjustment. Shaded blue region indicates baseline performance ± standard error of the mean (SEM) for a model with no predictors (intercept-only model where the predicted value was set to the mean synapse number for the sample). **B** Change in prediction error for each model, after DPOAE adjustment, relative to the same model without DPOAE adjustment. **C** Change in prediction error for each model relative to the ABR_80_ model when matched for DPOAE adjustment (i.e., RAM_1000_ model adjusted for DPOAE threshold would be compared to ABR_80_ model adjusted for DPOAE threshold). For all panels, the RMSE was computed on data pooled across 16 and 32 kHz for mice in the young, aged after noise exposure, and aged groups. Markers indicate the RMSE of the corresponding model averaged across all repeats and folds, colors indicate the DPOAE adjustment applied, and error bars indicate the SEM of the RMSE across all repeats and folds. For B and C, dashed line indicates the reference model

**Fig. 7:**
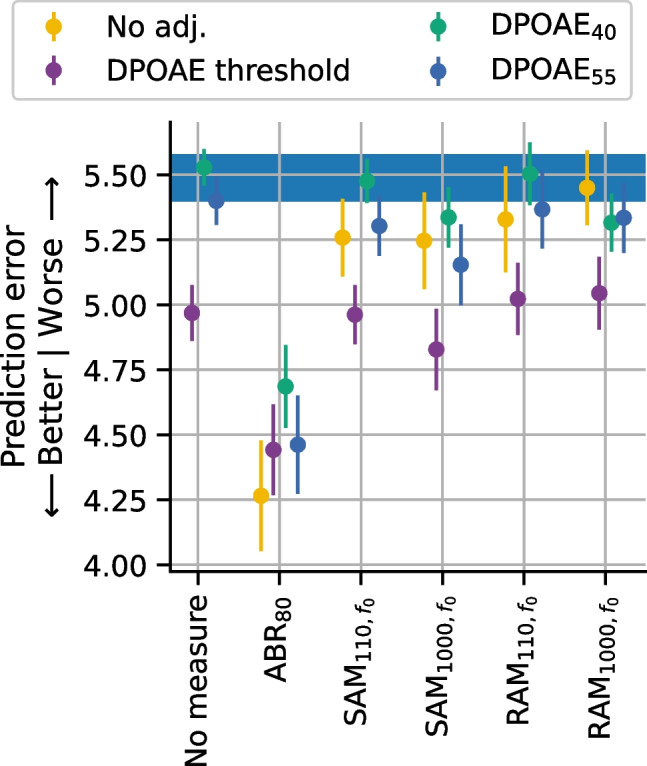
Relative ability of evoked potential models to predict synapse numbers in mice with focal synaptopathy. Root mean square error (RMSE) of each evoked potential model with and without DPOAE adjustment. Shaded blue region indicates baseline performance ± standard error of the mean (SEM) of a model with no predictors (intercept-only model where the predicted value was set to the mean synapse number for the sample). The RMSE was computed on data pooled across 16 and 32 kHz for mice in the acute noise exposed group. Markers indicate the RMSE of the corresponding model averaged across all repeats and folds, colors indicate the DPOAE adjustment applied, and error bars indicate the SEM of the RMSE across all repeats and folds

Since correlations simply capture linear relationships between variables, the prediction accuracy of each model was quantified using the root mean squared error (RMSE). The RMSE values indicate the prediction error in terms of number of synapses, with smaller values indicating better predictive power. RMSE was computed separately for each repeat and fold and then the mean and standard error were computed across all repeats and folds to give the pointwise estimate and standard error of the expected RMSE for future data. Baseline performance was assessed using an intercept-only model in which the predicted synapse count was the average synapse count for the entire dataset (blue bar in Figs. 6A and 7). The ability of the models to predict synapse numbers differed based on whether the configuration of synapse loss was focal or broad. For this reason, prediction accuracy was evaluated separately for these two types of synaptopathy configurations.

### Evaluation of Prediction Accuracy in Cases of No Synaptopathy or Broad Synaptopathy

In cases of no synaptopathy or broad synaptopathy (i.e., when the acute noise exposed group with focal synaptopathy was excluded), all models incorporating DPOAE and/or evoked potential data performed better than baseline when predicting the number of synapses (Fig. 6A). Models incorporating only a DPOAE measure performed similarly to models that included DPOAE-adjusted SAM_110_ or RAM_110_. Prediction performance for the ABR_80_ and DPOAE-adjusted SAM_1000_ models was somewhat improved over the DPOAE-only model. Overall, the DPOAE-adjusted RAM_1000_ models had the best prediction performance. The impact of the DPOAE adjustments varied across the evoked potential models. To quantify the effect of adjusting for DPOAEs, the difference in prediction error for each evoked potential model was calculated with or without DPOAE adjustment (Fig. 6B). DPOAE adjustment improved the predictive performance of models for each evoked potential measure, with the greatest impact on the SAM_110_ (0.56 to 0.77 improvement) and SAM_1000_ (0.53 to 0.98 improvement) models. The impact of DPOAE adjustment was intermediate for the RAM_110_ (0.15 to 0.33 improvement) and RAM_1000_ (0.09 to 0.37 improvement) models. DPOAE adjustment only had marginal impact for ABR_80_ (0.06 to 0.10 improvement). Given that ABR wave 1 amplitude is the gold standard for assessing auditory nerve function, the predictive performance of each model was also compared with the ABR wave 1 amplitude model (Fig. 6C). For all comparisons, the DPOAE adjustment was matched with that used in the ABR model (i.e., the SAM_110_ model adjusted for DPOAE threshold was compared with the ABR_80_ model adjusted for DPOAE threshold). Compared to ABR_80_, the prediction error was worse for the DPOAE-only (0.17 to 0.41 worse), SAM_110_ (0.21 to 0.88 worse), and RAM_110_ (0.14 to 0.39 worse) models, regardless of which method of DPOAE adjustment was used. In contrast, the SAM_1000_ model performed similarly to the ABR_80_ model (− 0.26 to 0.63 worse), but only when adjusted for DPOAEs. The RAM_1000_ model outperformed the ABR_80_ (0.16 to 0.43 better) regardless of DPOAE adjustment.

### Evaluation of Prediction Accuracy in Cases of Focal Synaptopathy

Figure 7 shows the prediction error of different DPOAE and evoked potential models when predicting synapse numbers for the acute noise exposed mice with focal synaptopathy. The ABR_80_ model clearly performed better than the other models regardless of whether a DPOAE adjustment was included. However, the prediction error of the ABR_80_ model (~ 4.5 synapses) was considerably poorer than the prediction error across all the models when evaluated in mice with broad synaptopathy (~ 3.5 synapses or less; Fig. 6A). In mice with focal synaptopathy, all the EFR models performed only slightly better than baseline and only when adjusted for DPOAE threshold. However, the DPOAE-adjusted EFR models did not outperform the matched DPOAE-only models. This indicates that the synapse predictions for focal synaptopathy generated by the EFR models are primarily driven by the inclusion of the DPOAE adjustment and that the EFR measurements likely did not contribute to the predictions.

### Comparison of Prediction Accuracy Across Models

As an additional complementary test of model performance, we fit each model to the full dataset without cross-validation and calculated the AICc. The difference in AICc between each model and the best-performing model was calculated (ΔAICc) and interpreted following the guidelines proposed by Burnham and Anderson [34]. Comparisons were made across the full set of models (Tables 1 and 2). AICc results indicate that ABR_80_ corrected for DPOAE_40_ is the best predictor of synapse number when considering mice with both broad and focal synaptopathy (shown in Table 1 by a dash). However, the ABR_80_ models that used other forms of DPOAE adjustment or did not adjust for DPOAEs performed similarly, as indicated by ΔAICc ≤ 2.62.

**Table 1:** ΔAICc values for each single evoked potential model compared to the best performing overall model in all mice. ΔAICc is computed using all mice in the study. The best performing model is indicated by a dash. Models with ΔAICc values ≤ 2 are considered comparable to the best performing model whereas models with ΔAICc values > 10 are considered to have poorer performance than the best performing model. Models with ΔAICc between 2 and 10 should not be rejected outright even though the evidence suggests that the model with a lower AICc is better

Measure	No adjustment	DPOAE threshold	DPOAE_40_	DPOAE_55_
None	59.65	19.51	25.92	35.28
ABR_80_	1.96	0.37	-	2.62
SAM_110_	53.72	21.59	27.56	35.69
SAM_1000_	40.29	12.8	15.1	24.81
RAM_110_	45.61	21.59	26.9	35.04
RAM_1000_	22.28	5.17	7.57	15.32

**Table 2:** ΔAICc values for each single evoked potential model compared to the best performing overall model in mice with no synaptopathy or broad synaptopathy. ΔAICc is computed after excluding the acute noise exposed group. The best performing model is indicated by a dash

Measure	No DPOAE adjustment	DPOAE threshold	DPOAE_40_	DPOAE_55_
None	86.73	42.51	37.62	56.11
ABR_80_	37.36	32.23	27.9	35.13
SAM_110_	83.05	44.18	39.54	57.92
SAM_1000_	59.62	27.41	19.29	37.91
RAM_110_	71.71	44.61	39.59	56.4
RAM_1000_	25.63	6.54	-	14.19

Given the poor performance of the evoked potential models in predicting focal synaptopathy, a separate AICc analysis was completed that excluded the mice from the acute noise exposed group (Table 2). These results indicate that RAM_1000_ adjusted for DPOAE_40_ outperformed all other evoked measures (shown in Table 2 by a dash). However, the RAM_1000_ adjusted for DPOAE threshold had a ΔAICc of 6.54, suggesting that it may perform similarly.

### Effects of Stimulus and Analysis Parameters on Synapse Prediction

**Fig. 8:**
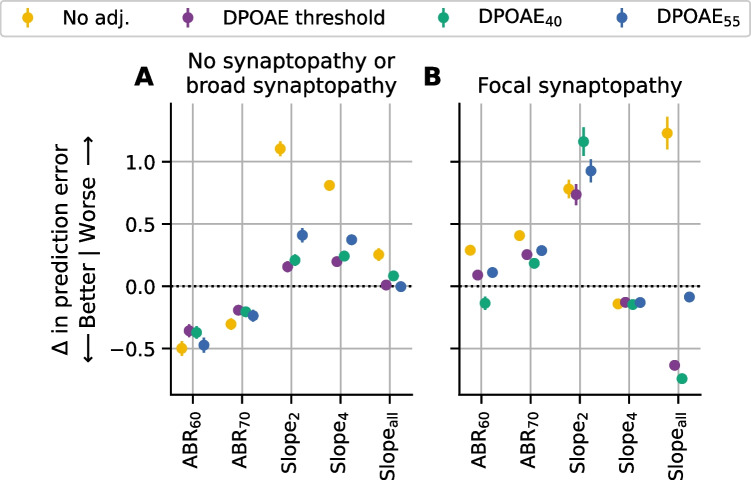
Impact of ABR stimulus level and ABR slope on synapse prediction performance. The change in prediction error for five alternative ABR models are shown relative to the ABR wave 1 amplitude model for 80 dB SPL (ABR_80_) when matched for DPOAE adjustment. Data is shown separately for mice with no/broad synaptopathy (**A**) or focal synaptopathy (**B**). Wave 1 amplitudes in response to lower stimulus levels (60 and 70 dB SPL; [ABR_60_ and ABR_70_]) were tested as well as three different ABR slope metrics. Slope was either calculated using the two highest stimulus levels (Slope_2_), the four highest stimulus levels (Slope_4_), or all stimulus levels (Slope_all_). The change in root mean square error (RMSE) was computed on data pooled across 16 and 32 kHz for mice in the young, aged noise exposed, and aged groups (**A**) or mice in the acute noise exposed group (**B**). Markers indicate the change in RMSE for the corresponding model relative to ABR_80_ averaged across all repeats and folds, colors indicate the DPOAE adjustment applied, and error bars indicate the standard error of the mean (SEM) of the change in RMSE across all repeats and folds. The dashed line in both panels indicates the reference model (ABR_80_)

High intensity level ABR stimuli are less frequency-specific than lower intensity stimuli due to the spread of excitation along the cochlear partition [38]. For this reason, we evaluated the relative synapse prediction ability for ABR wave 1 amplitude models using lower stimulus levels (60 and 70 dB SPL; [ABR_60_ and ABR_70_]). Both the ABR_60_ and ABR_70_ models had lower prediction errors than the ABR_80_ model in mice with no, or broad, synaptopathy (Fig. 8A). In contrast, neither the ABR_60_ or ABR_70_ models outperformed the ABR_80_ model in the mice with focal synaptopathy (Fig. 8B).

In some human studies of synaptopathy, it has been suggested that the growth rate (or slope) of ABR wave I amplitude may be a better indicator of synaptopathy than ABR wave I amplitude itself because it is a differential measure that minimizes the impacts of individual factors such as sex, head size, and electrode impedance that could affect wave I amplitude [e.g., 39]. To investigate this hypothesis, we evaluated the synapse prediction performance of the ABR amplitude models relative to several different ABR wave 1 amplitude slope models (slope calculated from all collected ABR stimulus levels [Slope_all_], slope calculated from the two highest stimulus levels [75 and 80 dB SPL; Slope_2_], and slope calculated from the four highest stimulus levels [65, 70, 75, and 80 dB SPL; Slope_4_]). For mice with broad synaptopathy (or no synaptopathy), none of the ABR slope models performed better than the wave 1 amplitude models (Fig. 8A). In contrast, in animals with focal synaptopathy, the model using the ABR slope calculated from all stimulus levels [Slope_all_] performed best, but only when corrected for either DPOAE threshold or DPOAE_40_ (Fig. 8B).

Given that methods of calculating EFR magnitude have varied across previous synaptopathy studies, the impact of different methods of calculating EFR magnitude (i.e., calculating EFR magnitude relative to the noise floor [dB SNR], including harmonics in the sum [*f*_0–4_], or using phase-locking value [PLV]) on the ability of the EFR models to predict synapse numbers was also evaluated (Supplemental Data Fig. 4). The most uniformly high performing analysis method across all EFR stimuli was the sum of the EFR magnitude at multiples of the modulation frequency without referencing to the noise floor (*f*_0–4_), although using only EFR magnitude at the modulation frequency (*f*_0_) was a close second. When corrected for DPOAEs, all the analysis methods performed similarly for the SAM_110_ stimulus that has typically been used in human studies of synaptopathy.

### Comparison of Models with Single Evoked Potential Measures and Models with Multiple Measures

**Fig. 9:**
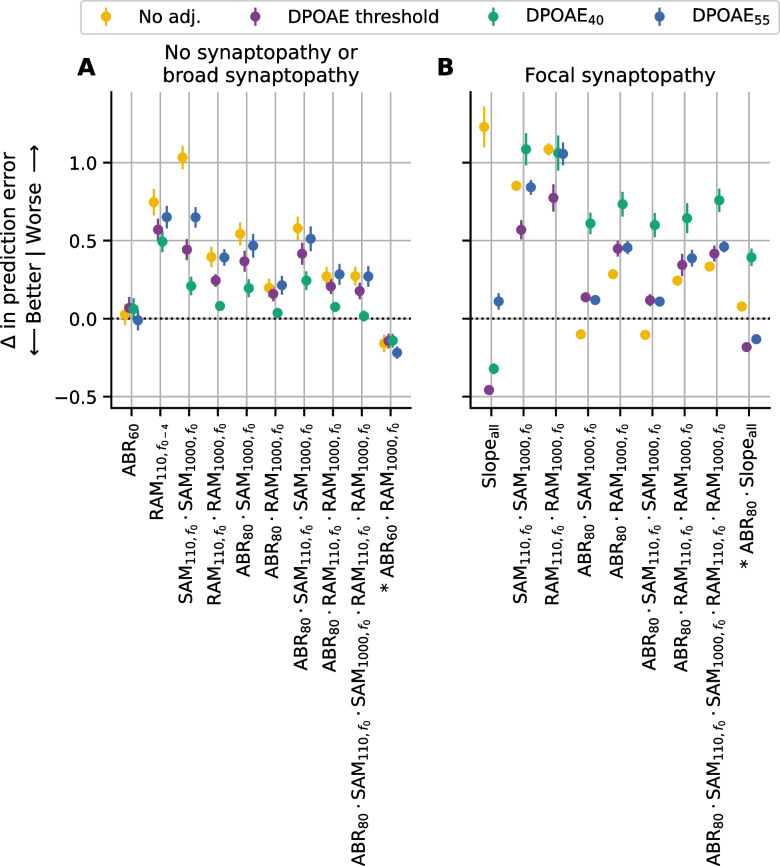
Impact on synapse prediction performance of incorporating multiple evoked potential measures. Change in prediction error for models incorporating various combinations of ABR, SAM EFR, and RAM EFR measures. Results are shown separately for mice with no/broad synaptopathy (**A**) relative to the best-performing single predictor model for that group (RAM_1000,*f0*_ corrected for DPOAE_40_) or focal synaptopathy (**B**) relative to the best-performing single predictor model for that group (ABR_80_ without DPOAE adjustment). The root mean square error (RMSE) was computed on data pooled across 16 and 32 kHz for mice in the young, aged noise exposed, and aged groups (**A**) or for mice in the acute noise exposed group (**B**). Markers indicate the change in RMSE of the corresponding model averaged across all repeats and folds, colors indicate the DPOAE adjustment applied, and error bars indicate the standard error of the mean (SEM) of the change in RMSE across all repeats and folds. Dashed line indicates the reference model (RAM_1000,*f0*_ corrected for DPOAE_40_ in (**A**), ABR_80_ without DPOAE adjustment in (**B**)). In (**A**), the ABR_60_ and RAM_110,*f0-4*_ models are also plotted because they showed additional improvements relative to similar versions of the model (Fig. 8, Supplemental Data Fig. 4). In (**B**), the Slope_all_ models are also plotted because they showed additional improvements relative to ABR wave 1 amplitude (Fig. 8B). Asterisks next to the model names indicate combinations that were added after review of the results shown in Fig. 8 and Supplemental Data Fig. 4. All other comparisons were planned prior to the start of the study

Since each evoked potential may encode different information about auditory nerve function, in mice with no synaptopathy or broad synaptopathy, we tested whether combining multiple evoked potential measures improves the ability to predict synapse numbers relative to the highest performing single evoked potential model (RAM_1000_ corrected for DPOAE_40_). Prior to beginning the study analyses, we established several combinations that we intended to test. None of these pre-determined combinations improved synapse prediction in mice with no synaptopathy or broad synaptopathy (Fig. 9A). After reviewing the results of our initial analysis, which suggested that the ABR_60_ model outperforms the ABR_80_ model in cases of broad synaptopathy (Fig. 8A), we added a new combination for testing (asterisks in Fig. 9). This new analysis indicated that the ABR_60_ · RAM_1000_ model yielded improvements in prediction error over both the ABR_60_-only (0.11 to 0.19 synapses better) and RAM_1000_-only (0.14 to 0.22 synapses better) models regardless of whether the single-measure models were adjusted for DPOAEs. Figure 9A also shows that the ABR_60_ model performed similarly to the RAM_1000_ model regardless of whether it was adjusted for DPOAEs (− 0.07 to 0.01 synapses better).

We performed a similar analysis in mice with focal synaptopathy, where we compared the models combining multiple evoked potential measures to the highest performing single evoked potential model (ABR_80_ with no DPOAE correction). First, we tested the same pre-determined combinations that were evaluated for broad synaptopathy. None of these pre-determined combinations resulted in improved synapse predictions in mice with focal synaptopathy relative to the best performing single-measure model (Fig. 9B). After reviewing the results of our initial analysis, which suggested that the Slope_all_ model outperforms the ABR_80_ model in cases of focal synaptopathy (Fig. 8B), we added two new combinations for testing (asterisks in Fig. 9). This new analysis revealed that a combined ABR_80_ · Slope_all_ model improved synapse prediction over the ABR_80_ model when adjusted for DPOAE threshold (0.18 synapses better) or DPOAE_55_ (0.13 synapses better), but the Slope_all_ model adjusted for DPOAE threshold had the best overall performance (0.46 synapses better).

We also fit each of these multiple-measure models to the full dataset without cross-validation and calculated the ΔAICc (Table 3). These results indicate that the ABR_60_ · RAM_1000_ model corrected for DPOAE threshold was the best overall performing model regardless of whether mice had broad or focal synaptopathy (shown in Table 3 by a dash). However, the ABR_60_ · RAM_1000_ models were relatively insensitive to the type of DPOAE adjustment (ΔAICc ≤ 1.93) and the ABR_60_-only models also performed relatively well (ΔAICc between 3.78 and 4.23).

**Table 3:** ΔAICc values for combination evoked potential models relative to the best performing model in all mice. ΔAICc is computed using all mice in the study. The best performing model is indicated by a dash. RAM_1000,*f0*_ and ABR_80_ are shown because they are, respectively, the best performing single-measure models for no/broad synaptopathy (Fig. 6) and focal synaptopathy (Fig. 7). The Slope_all_, ABR_60_, and RAM_110,*f0-4*_ models are shown because they showed additional improvements relative to similar versions of the model (Fig. 8, Supplemental Data Fig. 4)

Measures included	No adjustment	DPOAE threshold	DPOAE_40_	DPOAE_55_
RAM_1000_	66.83	49.73	52.13	59.87
ABR_80_	46.51	44.92	44.55	47.17
Slope_all_	64.75	33.73	30.99	40.34
ABR_60_	4.05	3.78	4.28	3.89
RAM_110,*f0-4*_	85.32	66.05	70.18	77.49
SAM_110_ SAM_1000_	83.12	59.35	61.77	70.8
RAM_110_ RAM_1000_	66.63	50.64	54.18	61.97
ABR_80_ SAM_1000_	47.97	46.08	45.45	48.54
ABR_80_ RAM_1000_	41.23	40.47	39.98	42.64
ABR_80_ SAM_110_ SAM_1000_	48.77	45.85	45.7	49.25
ABR_80_ RAM_110_ RAM_1000_	38.5	34.2	35.09	38.63
ABR_80_ SAM_110_ SAM_1000_ RAM_110_ RAM_1000_	42.04	37.78	38.66	41.9
ABR_60_ RAM_1000_	1.93	-	1.16	1.46
ABR_80_ Slope_all_	45.4	35.72	33.01	40.49

Given the particularly poor performance of the SAM and RAM EFR in predicting focal synaptopathy, a separate AICc analysis was completed that excluded the mice from the acute noise exposed group (Table 4). These results indicate that the ABR_60_ · RAM_1000_ model adjusted for DPOAE_55_ outperformed all other evoked measures for mice with no/broad synaptopathy (shown in Table 4 by a dash). However, all ABR_60_ · RAM_1000_ models, regardless of DPOAE adjustment, performed relatively well (ΔAICc ≤ 4.06).

**Table 4:** ΔAICc values for combination evoked potential models relative to the best performing model in mice with no synaptopathy or broad synaptopathy. ΔAICc is computed when excluding the acute noise exposed group. The best performing model is indicated by a dash. RAM_1000,f0_ is shown because it is the best performing single-measure models for no/broad synaptopathy (Fig. 6). The ABR_60_ and RAM_110,f0-4_ models are shown because they showed additional improvements relative to similar versions of the model (Fig. 8, Supplemental Data Fig. 4)

Measures included	No adjustment	DPOAE threshold	DPOAE_40_	DPOAE_55_
RAM_1000_	60.06	40.96	34.43	48.62
ABR_60_	22.84	24.09	23.8	21.12
RAM_110,*f0-4*_	98.87	78.77	72.97	87.77
SAM_110_ SAM_1000_	91.18	63.9	55.82	74
RAM_110_ RAM_1000_	58.69	42.24	36.13	50.78
ABR_80_ SAM_1000_	65.28	58.61	52.07	62.18
ABR_80_ RAM_1000_	44.7	40.04	34.43	43.19
ABR_80_ SAM_110_ SAM_1000_	67.45	60.1	53.62	64.27
ABR_80_ RAM_110_ RAM_1000_	46.66	39.83	34.79	44.37
ABR_80_ SAM_110_ SAM_1000_ RAM_110_ RAM_1000_	47.78	40.26	33.92	44.82
ABR_60_ RAM_1000_	4.06	3.02	3.63	-

## Discussion

Adjusting for DPOAEs generally improved the ability of the models to predict synapse numbers, particularly for the SAM EFR models. The differing impact of the DPOAE adjustment on the prediction performance of the SAM versus RAM EFR models is consistent with computational modeling studies suggesting that the sharp onset of the RAM EFR stimulus makes it less vulnerable to impacts from OHC dysfunction than the SAM EFR stimulus [15]. When assessing models that incorporated ABR wave 1 amplitude, it was not necessary to adjust for DPOAEs to yield models that performed well with the exception of the ABR slope models (Figs. 6, 7, 8, and 9). This suggests that in human studies of synaptopathy, it may not be necessary to statistically adjust for OHC function when using ABR wave I amplitude as an indicator of synaptopathy.

**Fig. 10:**
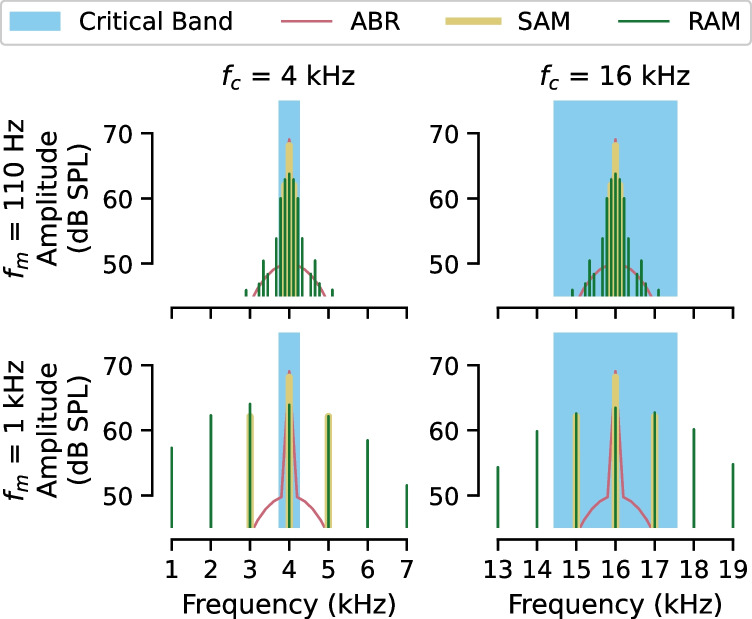
Spectral components of 4 kHz and 16 kHz ABR and EFR stimuli relative to the estimated critical bandwidth. For the RAM and SAM EFR stimuli, the spectral components are produced by modulation of a tonal carrier. Spectral components are positioned at ${f}_{c}\pm k\cdot {f}_{m}$ where ${f}_{c}$ is the carrier frequency, ${f}_{m}$ is the modulation frequency and *k* is 1 for SAM (yellow) or any number between 1 and ∞ for RAM (green). The shaded blue area indicates the critical bandwidth for ${f}_{c}$. For *f*_*c*_ = 16 kHz the dominant spectral components fall within the critical band, while for *f*_*c*_ = 4 kHz they do not. The ABR (pink) is a narrowband stimulus with the dominant magnitude at the stimulus frequency, staying within the critical band for both 4 kHz and 16 kHz stimuli. For comparison, all stimuli are matched in level at 70 dB SPL

In mice with broad synaptopathy (or no synaptopathy), the RAM_1000_ EFR model was more accurate at predicting synapse numbers than the other single evoked potential models, supporting findings of computational modeling that suggest that the RAM EFR is a better indicator of synaptopathy than the SAM EFR [15]. The better performance of the RAM_1000_ model as compared to the RAM_110_ model is consistent with animal studies demonstrating that the auditory nerve is the primary generator of the EFR at higher modulation frequencies [5, 6]. Unfortunately, it is unclear if a response to the RAM_1000_ EFR can be obtained in humans. A key limitation when translating this work to humans is that the equivalent rectangular bandwidth depends on cochlear frequency. At carrier frequencies of 16 and 32 kHz, the equivalent rectangular bandwidth in mice is ~ 3 and 10 kHz, respectively [40]. In humans, comparable carrier frequencies might be 4 and 8 kHz, which translate to equivalent rectangular bandwidths of 0.5 and 1 kHz, respectively [41]. Given that the SAM and RAM EFR have sidebands at ${f}_{c}\pm k\cdot {f}_{m}$ where $k=1$ for SAM and $k=1\dots \infty$ for RAM (Fig. 10), some stimulus components may fall outside the critical bandwidth of the cochlear filter. For example, for a 4 kHz EFR carrier frequency modulated at 1000 Hz, the sidebands fall outside the equivalent rectangular bandwidth for 4 kHz, while the sidebands for a 16 kHz EFR carrier modulated at 1000 Hz do not fall outside the equivalent rectangular bandwidth for 16 kHz. Despite this caveat, McHaney et al. [14] demonstrated that the SAM EFR using a 3 kHz carrier with a modulation frequency of 1024 Hz can be obtained in young adults with normal hearing using an ear canal electrode (tiptrode). However, questions regarding the interaction between EFR magnitude and critical bandwidth remain. Thus, future research should investigate the feasibility of collecting the RAM_1000_ EFR in humans and test whether the RAM_1000_ EFR remains a superior predictor of synapse number even in animal models with low frequency hearing.

The synapse prediction performance of the various ABR wave 1 amplitude models indicate that a model using a stimulus level of 60 dB SPL [ABR_60_] performed best for broad synaptopathy, with another lower level ABR stimulus model [ABR_70_] also resulting in better synapse predictions than the ABR_80_ model. This is consistent with the results of Lopez-Poveda and Barrios [42] suggesting that coding of low intensity stimuli will be more negatively impacted by the stochastic undersampling associated with cochlear deafferentation than high intensity stimuli. Previous human studies of synaptopathy have typically used high ABR stimulus levels [reviewed in 43], partly to ensure that wave I amplitude can be identified in all participants. Future studies may want to use the lowest ABR stimulus level that still allows for reliable measurement of wave I amplitude even if it requires increasing the number of averages and/or using electrode configurations (i.e., tiptrodes) that improve the signal-to-noise ratio.

The evaluation of the synapse prediction performance of ABR slope suggests that in cases of focal synaptopathy, ABR slope may be a better predictor of synapse number than ABR wave 1 amplitude. In focal synaptopathy, regions of the cochlea that fall outside the tonotopic extent of the noise-induced synaptic damage may respond to high level but not low level ABR stimuli. This would lead to steeper growth of ABR wave 1 amplitude as compared to ears with broad synaptopathy. Further, stochastic undersampling of the auditory signal results in greater degradation of stimulus fidelity at low stimulus levels as compared to high stimulus levels [44]. In other words, there is greater variance in the first spike latency across the population of surviving auditory nerve fibers when using a low intensity stimulus than when using a high intensity stimulus, which will result in smaller ABR wave 1 amplitudes. However, restricting the computation of ABR wave 1 slope to only the highest stimulus levels resulted in poorer model performance than using ABR wave 1 amplitude. This is problematic because calculating ABR slope with a large span of stimulus levels may be challenging in humans. Collecting ABR wave I amplitudes over such a large span of stimulus levels in humans is unlikely to be feasible, and in most cases, it will be unclear prior to testing whether a patient has focal versus broad synaptopathy. This suggests that ABR wave 1/I amplitude may be a better general-purpose metric to use for synapse prediction than ABR slope.

Although other EFR processing approaches performed well for some EFR metrics, models using the sum of the absolute magnitude at *f*_0_–*f*_4_ were consistently among the best performing of both the SAM and RAM EFR models regardless of modulation frequency. This suggests that future EFR studies should consider using this processing approach. However, there was little difference among the different approaches for the SAM EFR modulated at 110 Hz, suggesting that the results of previous human studies that used different EFR processing approaches would likely not be impacted by the type of processing used. Given that the noise floor reflects other factors (e.g., arousal state, electrical interference, etc.), incorporating the noise floor into EFR magnitude calculations may increase measurement error.

The improved synapse prediction for broad synaptopathy resulting from combining RAM_1000_ EFR and ABR_60_ wave 1 amplitude in the same model compared to using them as single predictors suggests that these measures capture complementary information about synaptopathy. While both the ABR and RAM_1000_ EFR are primarily driven by auditory nerve activity, they may differentially reflect the contributions of SR subgroups [45]. ABR wave 1 amplitude, measured with an interleaved approach to minimize adaptation, predominantly reflects the initial, onset-driven firing of auditory nerve fibers, a response characteristic of high SR fibers. A major shortcoming of ABR wave 1 amplitude is its inability to detect loss of low SR fibers [7]. While the RAM EFR has a steep rise time that will also trigger an onset response dominated by high SR fibers, the sustained nature of the RAM EFR also probes the adapted, steady-state firing and phase-locking abilities of the nerve fibers, a feature more typical of low SR fibers [46]. Computational modeling suggests that the RAM EFR should be sensitive to the loss of low SR fibers [18]. Thus, the ABR and RAM EFR likely provide distinct windows into the functional contributions of different auditory nerve fiber populations. However, any additional improvements in prediction error must be considered in the context of the additional testing time required for collecting the extra stimuli versus the size of the improvement in prediction error (expressed as a number of synapses per IHC) relative to the overall number of cochlear afferents. For example, the improvement in prediction error for cases of no/broad synaptopathy when using the combined ABR_60_ and RAM_1000_ model versus the RAM_1000_ model was approximately 0.2 synapses. Neonates have an average of 15 synapses per IHC at the peak of the synaptogram [3], indicating that 0.2 synapses represents approximately 3% of the total expected number of synapses.

Of all the evoked potential models, only the ABR wave 1 amplitude models performed better than the intercept-only model at predicting the number of synapses in mice with focal synaptopathy. However, even the ABR models performed poorly, with prediction errors of approximately four synapses (versus approximately two synapses for the RAM_1000_ model in cases of no synaptopathy or broad synaptopathy). One interpretation is that this is due to a lack of frequency specificity of the ABR at high stimulus levels. However, the fact that the ABR_60_ model did not outperform the ABR_80_ model at predicting focal synaptopathy (Fig. 8B) does not support this view. The poor performance of the ABR wave 1 amplitude models in predicting focal synaptopathy is somewhat surprising given that much of the previous work on the relationship between ABR wave 1 amplitude and synaptopathy was done in animal models of focal noise-induced synaptopathy [e.g., 1]. However, this result is consistent with a previous attempt to predict the number of synapses per IHC from ABR wave 1 amplitude and DPOAE levels using a partial least squares regression model [47], which also overestimated the number of synapses in mice with focal synaptopathy. In contrast to the results of the current study, a previous study of mice with noise-induced synaptopathy showed a linear relationship between mean ABR wave 1 amplitude and mean number of synapses per IHC in groups of mice with temporary threshold shifts and in groups of mice with permanent threshold shifts after adjustment for auditory thresholds [48]. However, the noise exposures in that study resulted in a broader configuration of synaptopathy with a minimum of 20% synaptic loss at 16 kHz, while in the current study the acute noise exposed mice did not have synaptic loss at 16 kHz compared to the young mice. In this study, the poorer performance of the RAM_1000_ EFR model compared to the ABR model when predicting focal synaptopathy may be due to the broader range of spectral components in the RAM stimulus (Fig. 10), which will drive regions of the cochlea outside the focal lesion. In addition, computational modeling suggests that at the high stimulus levels typically used in humans (70–80 dB SPL), the SAM EFR is dominated by the response from off frequency auditory nerve fibers, limiting the frequency specificity of the response [11, 49]. A similar lack of frequency specificity has been demonstrated for the RAM EFR [18]. This may limit the ability of both EFR measures to predict focal synaptopathy. Further work with a larger dataset of mice with varying degrees of focal synaptopathy may offer insights that can be used to fine-tune evoked potential model predictions for focal synaptopathy.

While the evoked potential models failed to accurately predict focal synaptopathy in the current study, this may not preclude diagnosis of human synaptopathy. Synapse counts from human temporal bones do not show any evidence of focal, or frequency-specific, synaptopathy [3, 4]. Even if focal synaptopathy does occur immediately following noise exposure in humans, it likely expands into broad synaptopathy over long time periods similar to what is observed in mice that are noise exposed and then aged [Fig. 2C; 19].

In conclusion, in mice with no synaptopathy or broad synaptopathy, the RAM_1000_ EFR model adjusted for DPOAE_40_ resulted in the best prediction of synapse numbers out of all the single evoked potential models, while a model that combined RAM_1000_ EFR and ABR_60_ wave 1 amplitude with the DPOAE_55_ adjustment provided the best overall performance out of all the predictive models that were evaluated. This suggests that future studies of synaptopathy should measure the RAM_1000_ EFR, ABR wave 1/I amplitude, and DPOAEs. However, concerns regarding the interaction between the critical bandwidth and the spectral components of the EFR stimuli require further study to establish whether the RAM_1000_ EFR remains a strong predictor of cochlear synapse number even at the low carrier frequencies needed to obtain this measure in humans. If the RAM_1000_ EFR turns out not to be a viable metric for use in humans, the results of this study suggest that ABR wave I amplitude, particularly for lower stimulus levels, is also a strong measure of synaptopathy. None of the evoked potential models performed well at predicting focal synaptopathy, although the ABR slope (from ABR threshold to 80 dB SPL) and ABR_80_ models performed the best. Additional work will be necessary to identify physiological measures that can accurately predict focal synaptopathy.

## Supplementary Information

Below is the link to the electronic supplementary material.

ESM 1 (DOCX 144 KB)

## Data Availability

The data supporting the findings of this study are openly available in Zenodo (10.5281/zenodo.17593142).

## Code Accessibility

All custom-written software used for stimulus generation, data acquisition, and analysis are available on GitHub under permissive licenses (MIT, BSD 3-clause, etc.). See citations for details.

## Declarations

### Disclaimer

The opinions and assertions presented are private views of the authors and are not to be construed as official or as necessarily reflecting the views of the Department of Veterans Affairs or the National Institutes of Health.
