# Supplementary material for: Predicting Cochlear Synaptopathy in Mice with Varying Degrees of Outer Hair Cell Dysfunction Using Auditory Evoked Potentials
Source: J Assoc Res Otolaryngol. 2025 Dec 13;27(1):61–81. doi: 10.1007/s10162-025-01015-x (PMC12949156; doi:10.1007/s10162-025-01015-x)
Supplement: Supplementary file 1 — (DOCX 144 KB) [file 10162_2025_1015_MOESM1_ESM.pdf]

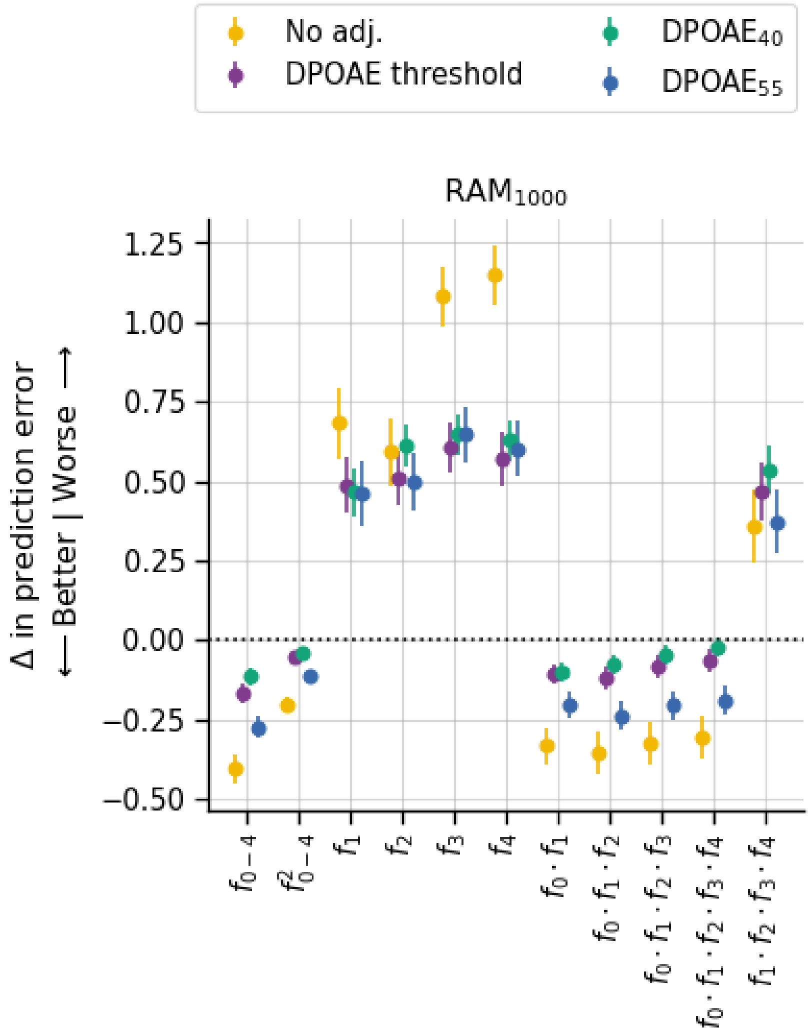

Supplemental Data Figure 1: Impact of calculating RAM_{1000} EFR magnitude/power on synapse prediction performance in mice with broad synaptopathy (or no synaptopathy). When reducing the first five multiples of the modulation frequency of the EFR to a single value, we can either sum the amplitude (f_{0-4}) or the power (f^2_{0-4}). However, summing may discard information that could be valuable for predicting synapse counts. An alternate approach is to treat each frequency component of the EFR as a separate predictor in the model EFR using the equation for testing p combinations of evoked potential measures (see Methods). By treating each frequency component (f_0 through f_4) as a separate predictor in the linear regression model, we can evaluate whether each harmonic, individually or in combination with other harmonics, contains information that facilitates synapse prediction. The x-axis labels indicate the

frequency components of the EFR that were included in the prediction model. EFR model results are plotted relative to the RAM_{1000} model where EFR magnitude was calculated using only the magnitude at the modulation frequency without correction for the noise floor (f_0, see Figure 1). Models were matched for DPOAE adjustment (e.g., f_{0-4} adjusted for DPOAE threshold was compared to f_0 adjusted for DPOAE threshold). The RMSE was computed on data pooled across 16 and 32 kHz for mice in the young, aged noise exposed, and aged groups. Markers indicate the change in RMSE of the corresponding model averaged across all repeats and folds, colors indicate the DPOAE adjustment applied, and error bars indicate the standard error of the mean (SEM) of the change in RMSE across all repeats and folds. Dashed line indicates the reference model ($RAM_{1000,f0}$).

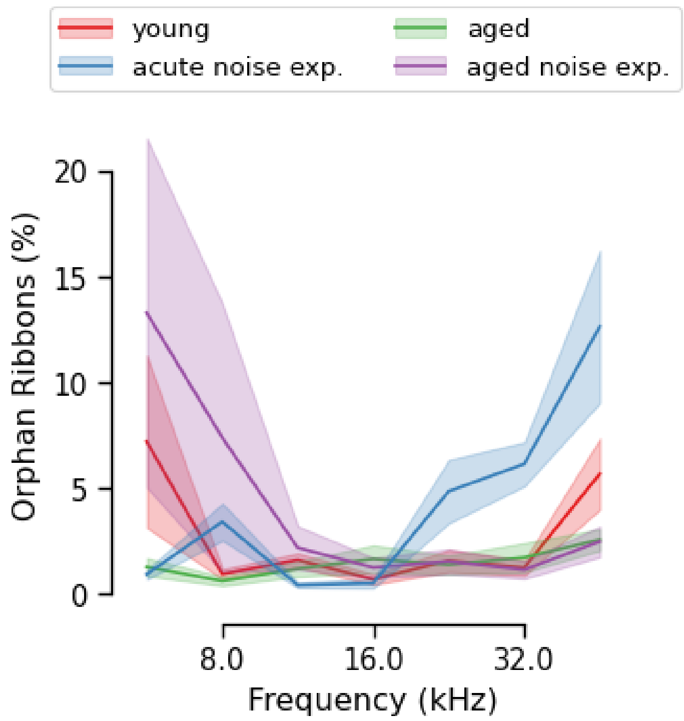

Supplemental Data Figure 2: The pattern of orphan ribbons varies between groups. Orphan ribbons are defined as synaptic ribbons (CtBP2 puncta) that do not have a closely-apposed post-synaptic receptor bouton (GluR2 patch). Acute noise-exposed mice had a greater proportion of orphan ribbons at frequencies within and above the noise-induced lesion whereas aged noise-exposed mice had the greatest proportion of orphan ribbons at low frequencies.

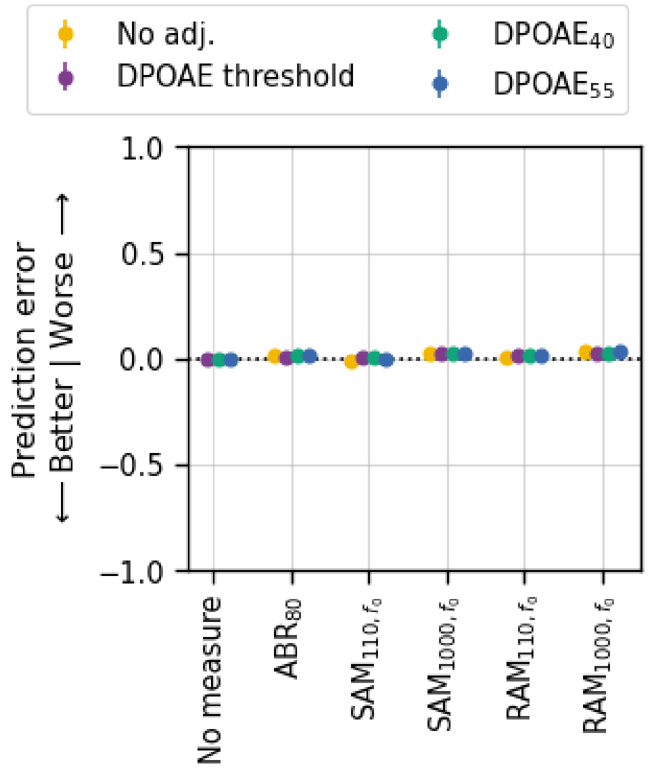

Supplemental Data Figure 3: Impact of adjusting for sex in prediction models. The impact on predictive power of including sex as a predictor was evaluated by constructing linear regression models based on various combinations of the evoked potential and DPOAE measures with or without sex as a predictor. Model results were matched for evoked measure and DPOAE adjustment and are plotted relative to the same model that does not include sex as a predictor (e.g., ABR_{80} adjusted for $DPOAE_{40}$ shows the change in prediction error when sex is included compared to the same model that does not include sex). The RMSE was computed on data pooled across 16 and 32 kHz for mice in the young, aged noise exposed, and aged groups. Markers indicate the change in RMSE of the corresponding model averaged across all repeats and folds, colors indicate the DPOAE adjustment applied, and error bars indicate the standard error of the mean (SEM) of the change in RMSE across all repeats and folds. Dashed line indicates the reference model (sex not included).

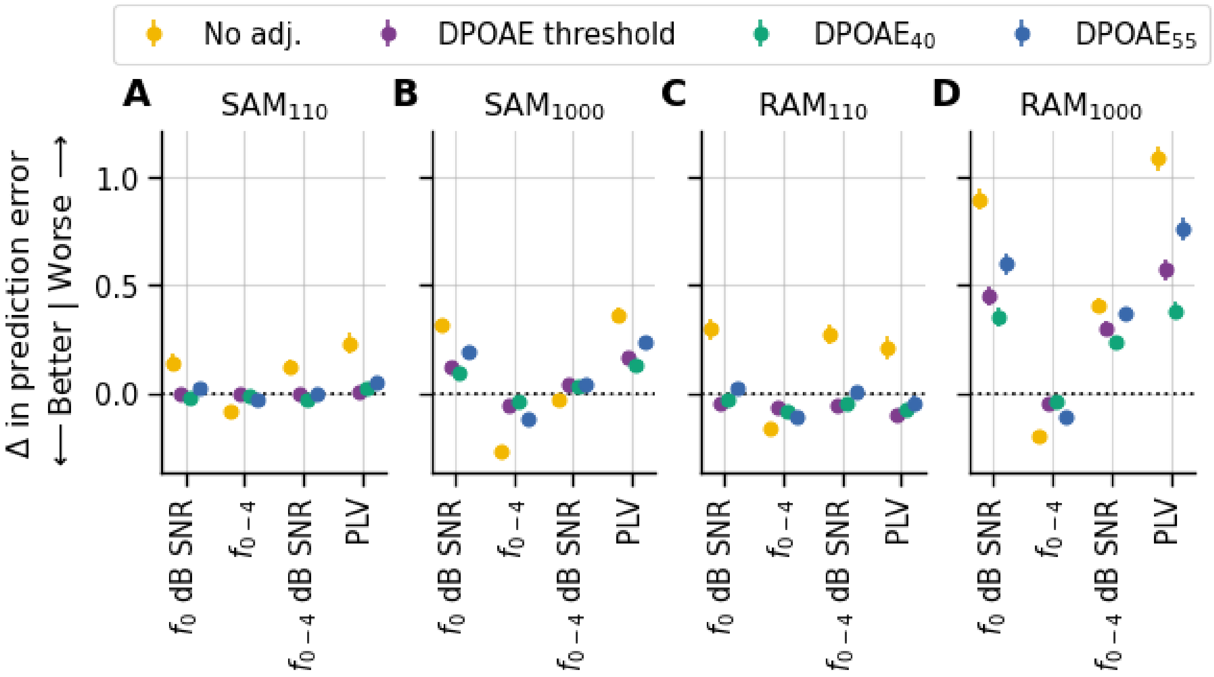

Supplemental Data Figure 4: Impact of EFR processing on synapse prediction performance in mice with broad synaptopathy (or no synaptopathy). EFR model results are plotted relative to the model where the same EFR measure was calculated using only the magnitude at the modulation frequency without correction for the noise floor (f_0, see Figure 1). Models were matched for DPOAE adjustment (e.g., SAM$_{110,f0-4}$ adjusted for DPOAE threshold was compared to SAM$_{110,f0}$ adjusted for DPOAE threshold). The change in root mean square error (RMSE) is plotted for the **(A)** SAM$_{110}$, **(B)** SAM$_{1000}$, **(C)** RAM$_{110}$, and **(D)** RAM$_{1000}$ models. The RMSE was computed on data pooled across 16 and 32 kHz for mice in the young, aged noise exposed, and aged groups. Markers indicate the change in RMSE of the corresponding model averaged across all repeats and folds, colors indicate the DPOAE adjustment applied, and error bars indicate the standard error of the mean (SEM) of the change in RMSE across all repeats and folds. Dashed line indicates the reference model (f_0). PLV = phase locking value, SNR = signal to noise ratio.
